# Polygon method: A systematic margin assessment for breast conservation

**DOI:** 10.1002/cam4.2211

**Published:** 2019-05-07

**Authors:** Shu Ichihara, Suzuko Moritani, Rieko Nishimura, Mikinao Oiwa, Takako Morita, Takako Hayashi, Aya Kato, Tokiko Endo, Akiko Kada, Noriko Ito, Tetsuo Kuroishi, Yasuyuki Sato

**Affiliations:** ^1^ National Hospital Organization Nagoya Medical Center Nagoya Japan; ^2^ Shiga University of Medical Science Otsu Japan; ^3^ National Hospital Organization Higashinagoya Hospital Nagoya Japan

**Keywords:** contralateral breast cancer, ipsilateral local recurrence, new primary, pancake phenomenon, true recurrence

## Abstract

**Background:**

Radiation therapy (RT) for women with ductal carcinoma in situ (DCIS) undergoing breast‐conserving surgery (BCS) may be overtreatment for some, especially for those in which DCIS is eradicated, and ipsilateral breast tumor recurrence (IBTR) risk approaches the contralateral breast cancer (CBC) level. The aim of this study was to clarify whether the polygon method, a new systematic method of en face (tangential, shaved) margin assessment, can identify a subset of DCIS that can be safely treated by BCS alone.

**Methods:**

A key tool of the polygon method is an adjustable mold that prevents the “pancake phenomenon” (flattening) of breast tissue after surgical removal so that the specimen is fixed in the shape of a polygonal prism. This preanalytical procedure enables us to command a panoramic view of entire en face margins 3‐5‐mm deep from the real peripheral cut surfaces. Competing risk analysis was used to quantify rates of IBTR and CBC and to evaluate risk factors.

**Results:**

From 2000 to 2013, we identified 146 DCIS patients undergoing BCS with a contralateral breast at risk. In 100 DCIS patients whose margin was negative by the polygon method, 5 IBTR (3 DCIS and 2 invasive ductal carcinoma [IDC]) and 10 CBC (6 DCIS and 4 IDC) cases were identified during a median follow‐up of 7.6 years (range, 0.9‐17.4). Five‐ and 10‐year cumulative incidence rates were 3.0% and 5.3% for IBTR, and 7.1% and 13.3% for CBC, respectively. Thus, patients with a negative margin consistently showed at least twofold lower IBTR than CBC despite omission of RT.

**Conclusions:**

Japanese women classified with a negative margin by the polygon method show a very low risk of IBTR and account for approximately half of CBC cases. In this subset of DCIS patients, additional RT is not beneficial.

## 1 INTRODUCTION

As a result of population‐based mammographic screening, an increasing number of women with small and unifocal ductal carcinoma in situ (DCIS) lesions undergo breast‐conserving surgery (BCS). At present, most guidelines recommend radiation therapy (RT) following BCS for DCIS based on randomized control trials comparing excision only with excision followed by RT; these studies indicate that the addition of RT halves the risk of ipsilateral breast tumor recurrence (IBTR).^1^, ^2^, ^3^, ^4^ However, RT for all women with DCIS may be overtreatment for some, especially for those in which the index carcinoma has been surgically eradicated.^5^ However, clear‐cut criteria for the selection of a subset of DCIS patients who could safely avoid RT have not been established.

Nineteen years ago, we adopted a new strategy of en face (shaved, tangential) margin assessment called the polygon method, incorporating peripheral vertical sectioning originally designed for cutaneous neoplasms.^6^ A key tool of the polygon method is an adjustable mold to prevent breast tissue from flattening after surgical removal so that the specimen is fixed in the shape of a polygonal prism.^7^, ^8^ This preanalytical procedure enables us to command a panoramic view of entire peripheral margins by shaving 3‐5 mm deep from the flat peripheral cut surfaces. Through this approach, the interphase between the critical peripheral boundaries of the excised tissue and the preserved tissue can be observed as continuous en face margins, whereas the interphase between the excised tissue and the pectoral muscle fascia (deep margin) and that between the excised tissue and the skin (superficial margin) are examined by conventional inked (perpendicular) margins. Thus, our strategy allows an assessment of the entire interphase between the removed and preserved tissue.

The aim of this study was to clarify whether the polygon method, a new systematic method of en face margin assessment, can identify a subset of DCIS that can be safely treated by BCS alone, at least in Japanese women. In this study, we hypothesized that if the margin evaluated by the polygon method is negative, true recurrence (TR) disappears because the index DCIS is eradicated. Namely, IBTR in patients with a negative margin as identified by the polygon method is solely new primaries (NP), that is, de novo malignancies arising from the residual terminal duct lobular units (TDLUs). If our hypothesis is true, the IBTR rate should be lower than the contralateral breast cancer (CBC) rate because both should simply reflect the distribution of normal TDLUs, which are fewer in number in the affected side than the healthy side after BCS. This is based on the same concept as the hypothesis that the high proportion of carcinomas arising in the upper outer quadrant of the breast is a reflection of the greater amount of TDLUs in this quadrant.^9^ Previous studies have usually been based on sections perpendicular to the outer surface of the specimen.^10^, ^11^, ^12^, ^13^, ^14^, ^15^, ^16^ To our knowledge, this is the first outcome study to explore the influence of complete en face (shaved, tangential) margin evaluation on local recurrence after BCS.^17^, ^18^, ^19^, ^20^

## 2 METHODS

### 2.1 Patients

All patients with DCIS who received BCS between 1 January 2000 and 31 December 2013 at Nagoya Medical Center were identified from the Breast Cancer Registry, which has been prospectively maintained since 1960. To identify DCIS patients with a contralateral breast at risk, we used an electronic medical record system to cross‐check patients. Patients were excluded if they had contralateral breast cancer prior to or synchronous with the diagnosis of DCIS, if they had mastectomies or if they underwent margin assessment other than the polygon method described here. Clinicopathological factors were collected based on the index DCIS, including age at diagnosis (≦49 or ≧50), menopausal status, family history, presentation (clinical or radiological), nuclear grade (low or high/intermediate), size (≦20 mm or ≧21 mm), ER, and HER2 status, and use of adjuvant treatment (RT, endocrine therapy) for the index DCIS. The study was retrospective and thus the treatment was not randomized. Treatment of individual cases was determined after a discussion between the physician and patient. Although patient's preferences are important in treatment selection, we recommend reexcision or mastectomy rather than RT for margin‐positive cases, especially for intermediate‐ or high‐grade DCIS that has more than 2 blocks with positive sites. It is also important to note that the polygon method is so sensitive that approximately half of positive cases by our method would be identified as negative by conventional methods. All patients with BCS were carefully followed up annually by ultrasound and mammography, and if necessary, magnetic resonance imaging (MRI). This study was approved by the Institutional Review Board.

### 2.2 Statistical analysis

Ipsilateral breast tumor recurrence, CBC, and death were considered as competing risks in this study. Therefore, Gray's test^21^ was used to quantify rates of IBTR and CBC and to evaluate the association of each factor with the cumulative incidence of IBTR or CBC. We could only perform a univariate analysis in the current study because the total number of events was too small for a multivariate analysis to obtain meaningful results. For DCIS patients treated with BCS and with excision specimen margins evaluated by the polygon method, the time (year) from definitive BCS for the initial DCIS to diagnosis of IBTR or CBC, including either DCIS or invasive breast cancer, was determined.

All statistical analyses were performed with EZR (Saitama Medical Center, Jichi Medical University, Saitama, Japan), which is a graphical user interface for R (The R Foundation for Statistical Computing, Vienna, Austria). More precisely, it is a modified version of R commander designed to add statistical functions frequently used in biostatistics.^22^

### 2.3 Pathological margin evaluation

**Figure 1:**
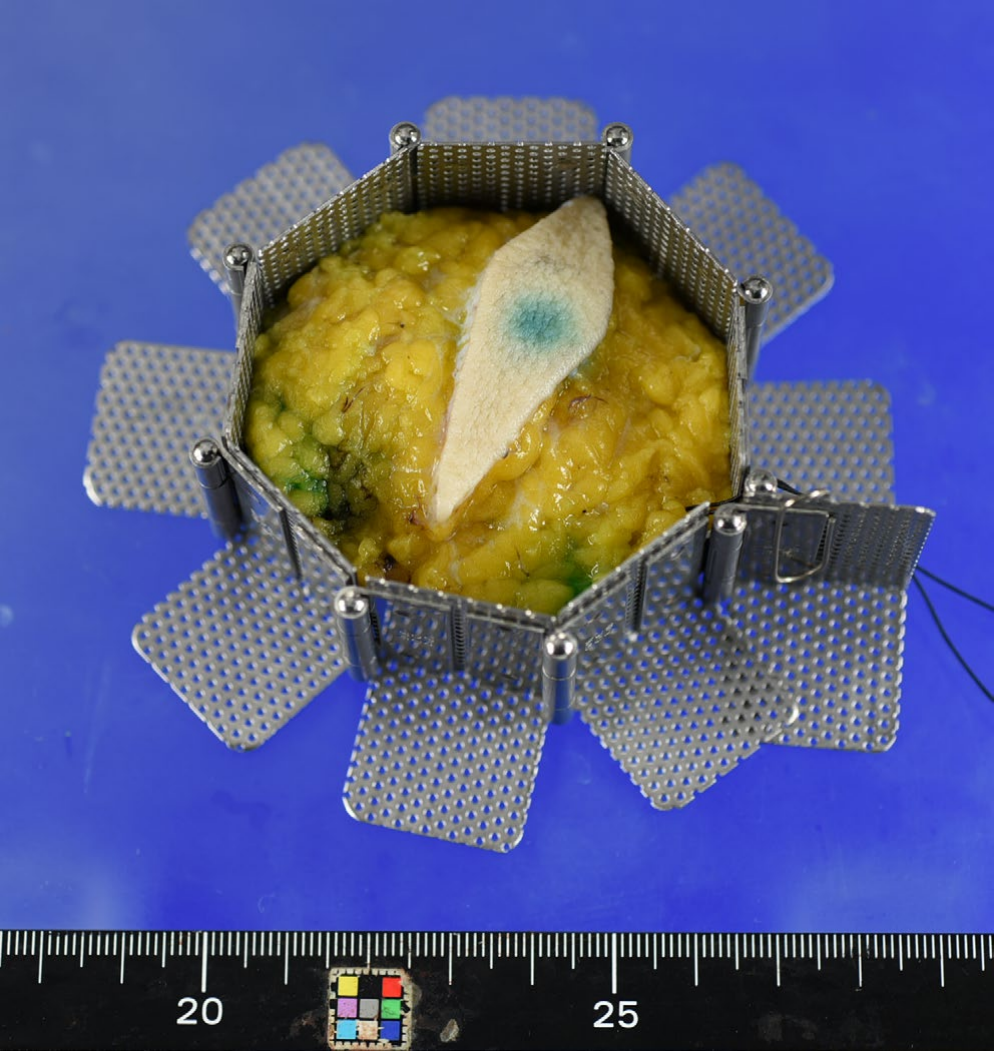
An adjustable mold to maintain the polygonal prism shape of the specimen. The mold is composed of rectangular‐shaped plates that are hinged together to change the angle between adjacent plates to form a polygonal prism for storage of excised tissue. The plates are made of punching metal to enable fixative formalin to pass through them and the size of each plate was calculated to fit the cassette for histological specimen preparation. (For inquiries concerning an adjustable mold (polygon mold®), please refer to PLM Co., Ltd. Toyoyama‐cho, Aichi, Japan (http://plm‐co.jp/WP))

The basic idea of our method for assessing the margin status of BCS was described for quadrantectomy in 2001^7^ and for wide local excision in 2003.^8^ Breast specimens resulting from wide local excision take the shape of a polygonal prism because surgeons tend to remove the whole lesion en bloc by vertical resection from the skin to the fascia of the greater pectoral muscle, namely, from the anatomical upper to lower limit. Therefore, we use a unique device to prevent deformity of the specimen after surgical removal. This device is an adjustable mold to maintain the polygonal prism shape of the specimen. The mold is composed of rectangular‐shaped plates that are hinged together to change the angle between adjacent plates to form a polygonal prism for storage of excised tissue (Figure 1). The plates are made of punching metal to enable fixative formalin to pass through them and the size of each plate was calculated to fit the cassette for histological specimen preparation. After 24 hours, the tissue maintains the shape of a polygonal prism following release from the mold. As a result, the boundaries of the lesion on the pectoral muscle fascia and the skin are examined by conventional inked (perpendicular) margins, whereas the boundaries between the removed and the preserved breast are examined by sensitive en face margins.

**Figure 2:**
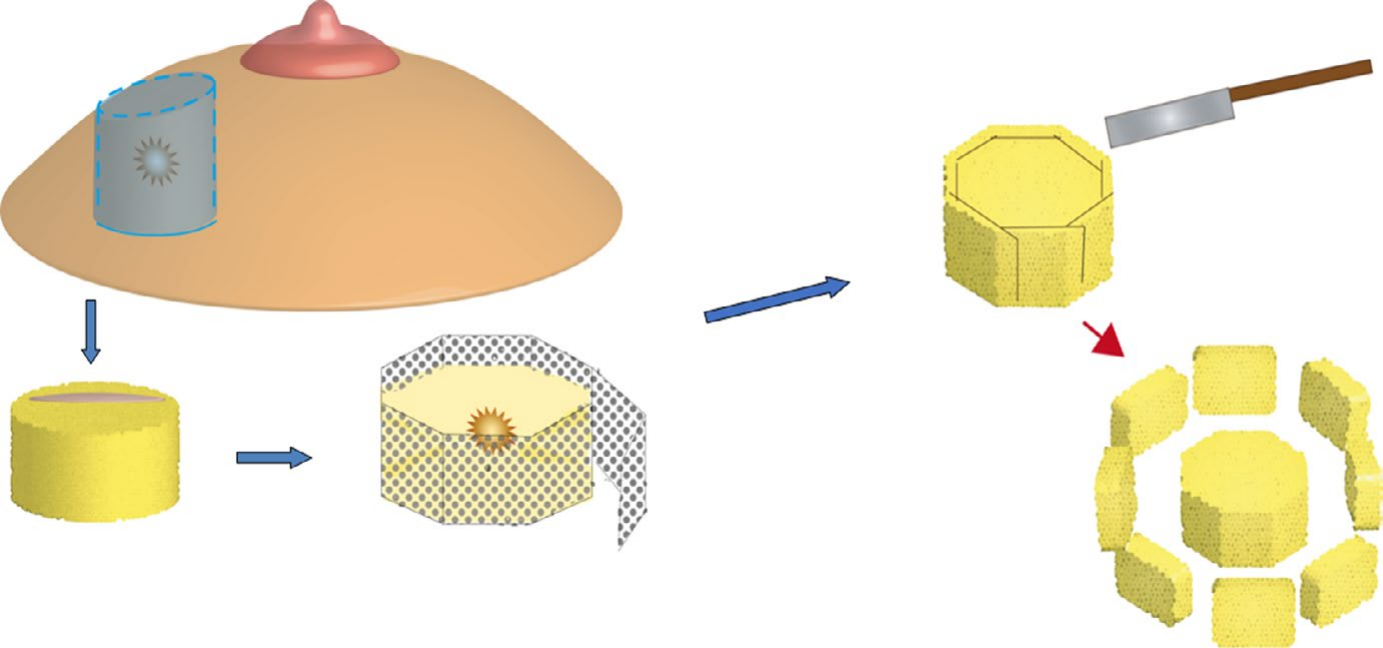
Flow chart illustrating the polygon method. To check the entire interphase between the resected and preserved breast tissue in wide local excision, we developed a precision margin assessment (the polygon method) incorporating the peripheral sectioning method originally designed for cutaneous neoplasms. The main tool for this method is an adjustable mold made of punching metal to prevent the pancake effect (flattening) of the specimen after surgical resection. After fixation in the adjustable mold, the specimen is in the shape of a polygonal prism. En face margins are cut to approximately 3‐4 mm in thickness. Histological sections are taken from the inner surface of the margins and observed by microscopy

En face margins are cut to approximately 3‐4 mm in thickness. Histological sections are taken from the inner surface of the shaved margins and observed by microscopy because the outer surface is irregular and often has crevasses or defects^23^ (Figure 2). The peripheral en face margin is considered negative if no carcinoma is present anywhere on the section from the inner surface of the shaved margins. If carcinoma is recognized on the inner surface, the margin is considered positive. The number of paraffin blocks with positive sites was recorded as these data can be used to quantify the impact of the positivity on the clinical outcome.

After obtaining peripheral en face margins from a polygonal prism‐shaped specimen, the rest of the specimen is usually processed by sequential slicing at intervals of 5 mm to exclude microinvasion as well as to evaluate the margin status of the 2 faces on the pectoral muscle fascia and that on the chest skin, the former of which are painted with ink. The pectoral muscle fascia or the skin margins are judged to be positive if the carcinoma cells are recognized on the ink, just as in the conventional inked margin method. The distribution of DCIS in the specimen is shown on a map for clinicians to facilitate image‐pathology correlation studies. This map is also useful for pathologists to evaluate whether the foci of atypical ductal hyperplasia (ADH) recognized at the margins represent either the periphery of the low‐grade DCIS or an isolated focus of ADH.

Histopathological diagnosis of DCIS and subdivision into low‐, intermediate‐, and high‐grade disease were performed according to the WHO classification of breast tumors.^24^ Encapsulated papillary carcinoma, solid papillary carcinoma in situ, and combined ductal and lobular carcinoma in situ were included in this study, whereas microinvasive carcinoma was excluded. ADH, lobular neoplasia, and flat epithelial atypia recognized at the margin were considered negative in this study.

## 3 RESULTS

Using the Breast Cancer Registry, we identified 176 Japanese women with DCIS treated by BCS between 1 January 2000 and 31 December 2013. Eighteen cases were excluded because they had contralateral breast cancer prior to or synchronous with the diagnosis of DCIS. Twelve cases were excluded because they underwent margin assessment other than the polygon method described here (n = 12). As a result, 146 DCIS (Stage 0) patients were analyzed in this study.

### 3.1 Surgical margin status by the polygon method

**Figure 3:**
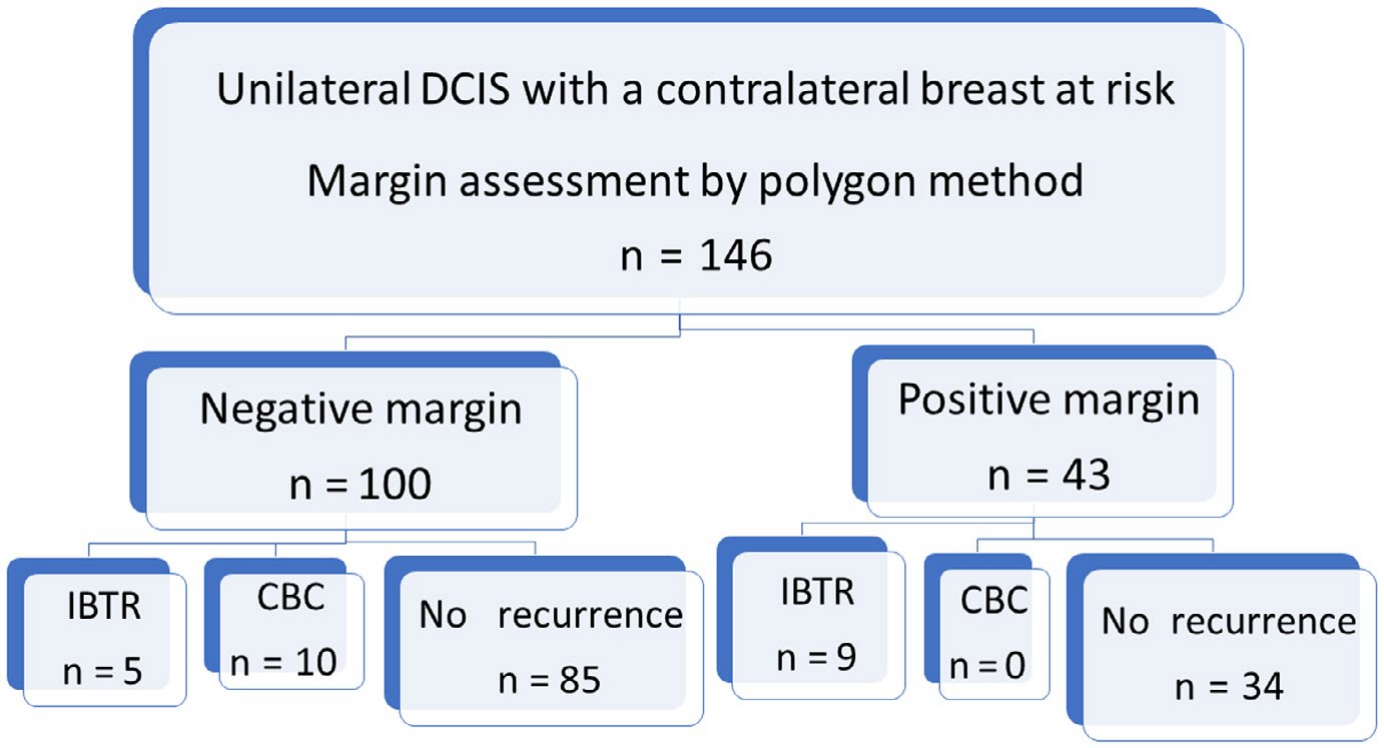
Flow chart. Using the Breast Cancer Registry prospectively maintained by Nagoya Medical Center, we retrieved 146 patients of DCIS (Stage 0) for analysis in this study. According to the polygon method, the margin status was negative in 100 cases and positive in 46 cases. Of the margin‐negative group, 5 developed IBTR (3 DCIS and 2 IDC) and 10 developed CBC (6 DCIS and 4 IDC) during a median follow‐up period of 7.6 y (range, 0.9‐17.4). CBC, contralateral breast cancer; DCIS, ductal carcinoma in situ; IBTR, ipsilateral breast tumor recurrence; IDC, invasive ductal carcinoma

**Figure 4:**
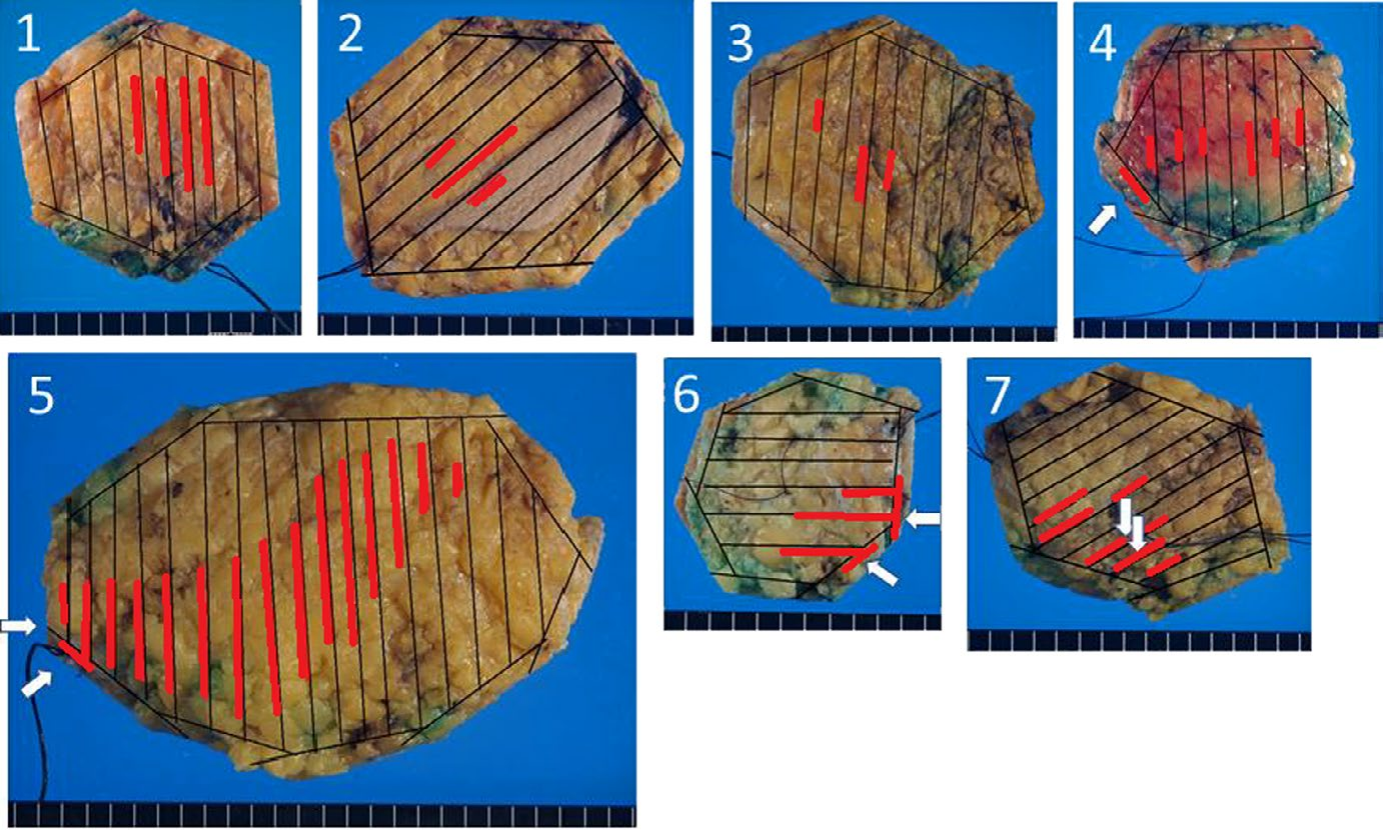
Examples of wide local excision specimens evaluated by the polygon method. In DCIS with positive margins, the distribution of DCIS (shown on the map as red bars) are well correlated with the positive sites (arrows) at the peripheral en face margins. 1. Low‐grade DCIS with a negative polygon margin. The polygon margin was negative and bread loaf slices also show a unifocal DCIS in the specimen. The patient developed invasive ipsilateral recurrence 8.3 y after surgery. 2. High‐grade DCIS with a negative polygon margin. The patient experienced ipsilateral DCIS one year after surgery. 3. Multifocal low‐grade DCIS with a negative polygon margin. The patient developed squamous carcinoma 0.8 y after surgery. 4. High‐grade DCIS with a positive margin in one block. The woman developed ipsilateral invasive recurrence 3.3 y after surgery. 5. High‐grade DCIS with a positive polygon margin at 2 blocks. The patient experienced ipsilateral in situ recurrence 2.1 y after surgery. 6. Intermediate‐grade DCIS with a positive polygon margin at 2 blocks. The woman had ipsilateral in situ recurrence 8 years after surgery. 7. High‐grade DCIS with a positive polygon margin in 2 blocks. This is the only case in this study in which the positive site was not the en face margin but the deep (pectoral muscle) margin. The patient developed ipsilateral in situ recurrence 3.2 y after surgery. DCIS, ductal carcinoma in situ

The surgical margin status of 146 cases of DCIS was determined by the polygon method: 100 cases were negative and 46 cases were positive (Figure 3). The vast majority (45/46) of margin positivity was due to the DCIS being located in the peripheral en face margins. In DCIS with positive margins, the distribution of DCIS shown in the map was well correlated with the positive sites at the peripheral en face margins (Figure 4). The number of paraffin blocks used per case ranged from 5 to 17 (median, 7) for peripheral shaved margin evaluation and from 4 to 49 (median, 12) for deep and superficial inked margin assessment as well as tumor characterization (Tables 1 and 2). In 80% of the cases, the number of blocks needed for the peripheral shaved margin evaluation were 8 or less. This implies that most of the specimens were fixed using pentagonal, hexagonal, heptagonal, or octagonal prism‐shaped molds. Except for 1 case with a positive margin, no radiotherapy was performed. Endocrine therapy was administered to 60 patients (41%). There were 7 recurrence‐unrelated deaths.

**Table 1:** Number of paraffin blocks used for peripheral en face margin assessment

Blocks (n)	Cases (n)
5	13
6	38
7	45
8	20
9	10
10	12
≧11	8
Total	146

Note

n, number.

**Table 2:** Number of paraffin blocks used for deep and superficial margin assessment and tumor characterization

Blocks (n)	Cases (n)
≦10	60
11‐20	59
21‐30	16
31‐40	6
≧41	5
Total	146

Note

n, number.

### 3.2 Demographic characteristics of the entire population, IBTR, and CBC

In the 100 patients with negative margins, 5 IBTR (3 DCIS and 2 invasive ductal carcinoma [IDC]) and 10 CBC (6 DCIS and 4 IDC) cases were identified during a median follow‐up of 7.6 years (range, 0.9‐17.4). In the 46 patients with positive margins, 3 patients underwent additional total mastectomies and 1 patient further wide local excision within 1 or 2 months following BCS. Of 43 margin‐positive patients, 9 developed IBTR (6 DCIS and 3 IDC). None of the margin‐positive group developed CBC.

In this study, IBTR was further classified into IBTR‐NP and IBTR‐TR based on the margin status. IBTR with a negative margin by the polygon method at the primary BCS was classified as IBTR‐NP (n = 5), which may arise from normal TDLUs. IBTR with a positive margin by the polygon method at the primary BCS was classified as IBTR‐TR (n = 9), occurring from residual in situ lesions of the initial DCIS. The index DCIS that developed IBTR‐NP included many low‐grade, ER‐positive DCIS, and only a few HER2‐positive DCIS. In contrast, the index DCIS that developed IBTR‐TR included many high‐grade, ER‐negative, and HER2‐positive DCIS cases. Thus, the DCIS that developed IBTR‐NP had much in common with the DCIS that developed CBC (n = 10). Demographic characteristics of the entire population, as well as those with IBTR‐NP, IBTR‐TR, and CBC, are summarized in Table 3.

**Table 3:** Demographic characteristics of the entire population (n = 146) and by ipsilateral tumor recurrence and contralateral breast cancer as the first subsequent breast event

		Total population (n = 146)		IBTR				CBC	
				IBTR‐NP (n = 5)		IBTR‐TR (n = 9)		(n = 10)	
		n	%	n	%	n	%	n	%
Age	≦49	62	42.4	4	80.0	6	66.7	4	40.0
	≧50	84	57.5	1	20.0	3	33.3	6	60.0
Menopausal status	No	96	65.8	3	60.0	6	66.7	9	90.0
	Yes	50	34.2	2	40.0	3	33.3	1	10.0
Family History	No	121	82.9	3	60.0	8	88.9	9	90.0
	Yes	23	15.8	2	40.0	1	11.1	1	10.0
	Unknown	2	1.4	0	0	0	0	0	0
Presentation	Clinical	81	55.4	5	100	8	88.9	4	40.0
	Radiological	58	39.7	0	0	1	11.1	5	50.0
	Unknown	7	4.8	0	0	0	0	1	10.0
Nuclear grade	Low	49	33.6	4	80.0	1	11.1	9	90.0
	High/intermediate	97	66.4	1	20.0	8	88.9	1	10.0
Size	≦20mm	91	55.5	3	60.0	2	22.2	9	90.0
	≧21mm	55	37.7	2	40.0	7	77.8	1	10.0
ER	+	114	78.1	4	80.0	5	55.6	8	80.0
	−	29	19.9	1	20.0	4	44.4	1	10.0
	Unknown	3	2.1	0	0	0	0	1	10.0
HER2	3+	22	15.1	1	20.0	4	44.4	0	0
	2+	15	10.3	0	0	1	11.1	2	20.0
	1+	41	59.9	1	20.0	1	11.1	5	50.0
	0	45	65.7	2	40.0	3	33.3	1	10.0
	Unknown	23	15.8	1	20.0	0	0	2	20.0
Radiation	No	145	99.3	5	100	9	100	10	100
	Yes	1	0.7	0	0	0	0	0	0
Endocrine therapy	No	86	58.9	5	100	4	44.4	7	70.0
	Yes	60	41.1	0	0	5	55.5	3	30.0

Abbreviations: DCIS, ductal carcinoma in situ; ER, estrogen receptor; HER2, human epidermal growth factor receptor; IBTR‐NP, ipsilateral breast tumor recurrence‐new primary; IBTR‐TR, ipsilateral breast tumor recurrence‐true recurrence.

### 3.3 Competing risk analysis of IBTR versus CBC

**Figure 5:**
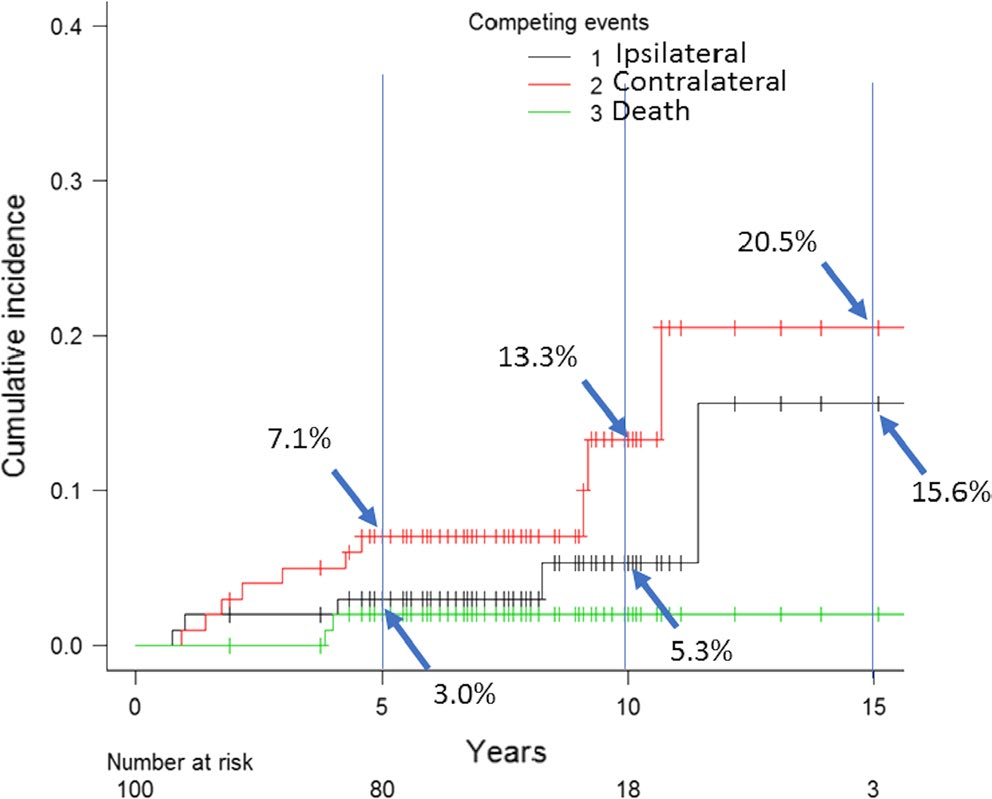
Cumulative incidence of ipsilateral and contralateral carcinoma. Competing risk analysis of 100 DCIS patients with negative margins demonstrated 5‐ and 10‐year IBTR rates of 3.0% and 5.3%, respectively, compared with 7.1% and 13.3%, respectively, for CBC. CBC, contralateral breast cancer; DCIS, ductal carcinoma in situ; IBTR, ipsilateral breast tumor recurrence

Competing risk analysis revealed 5‐year and 10‐year IBTR rates of 6.2 and 9.8%, respectively, compared with 4.9 and 8.5%, respectively, for CBC (Table 4). Median follow‐up was 7.6 years (range, 0.9‐17.4). On univariate competing risk analysis, the factors significantly associated with risk of IBTR were young age at diagnosis (≦49), presentation (clinical), positive margin status by the polygon method, large size of DCIS (≧21 mm) and 3+ HER2 status. Of note, for the DCIS patients with negative margins (n = 100), 5‐ and 10‐year cumulative incidence rates were 3.0% and 5.3% for IBTR, and 7.1% and 13.3% for CBC, respectively. (Figure 5). Thus, patients with a negative margin consistently showed IBTR of at least twofold lower than CBC despite RT being omitted. The factors significantly associated with risk of CBC were negative margin status by the polygon method, small size of DCIS (≦20 mm), and low nuclear grade.

**Table 4:** Univariate competing risk analysis of 5‐year and 10‐year cumulative incidence rates for ipsilateral breast tumor recurrence and contralateral breast cancer by clinicopathological characteristics at initial diagnosis of DCIS

	Ipsilateral breast tumor recurrence			Contralateral breast carcinoma		
	5‐year risk (%)	10‐year risk (%)	*P*‐Value^a^	5‐year risk (%)	10‐year risk (%)	*P*‐Value^b^
Entire population	6.2	9.8		4.9	8.5	
Age						
≦49	9.9	16.1	0.02	4.9	9.0	0.84
≧50	3.6	4.9		4.8	8.0	
Presentation						
Radiological	1.7	1.7	0.02	6.9	6.9	0.43
Clinical	10.0	15.9		2.5	8.1	
Margin status by polygon method						
Negative	3.0	5.3	0.01	7.1	13.3	0.01
Positive	13.2	19.3		0	0	
Size of DCIS						
≦20 mm	4.4	6.7	0.04	6.6	12.5	0.05
≧21 mm	9.3	15.2		1.9	1.9	
Nuclear grade						
Low	4.4	7.6	0.37	7.3	15.6	0.03
High/intermediate	8.0	12.0		2.6	2.6	
ER						
Negative	17.2	17.2	0.18	3.4	3.4	0.45
Positive	3.5	8.2		4.4	8.9	
HER2						
0‐2+	4.0	9.7	0.04	6.0	8.7	0.40
3+	22.7	22.7		0	0	
Endocrine therapy						
No	8.2	11.2	0.36	8.2	8.2	0.21
Yes	3.4	7.9		0	5.9	

Abbreviations: DCIS, ductal carcinoma in situ; ER, estrogen receptor; HER2, human epidermal growth factor receptor 2.

a *P*‐Value for each covariate for risk of IBTR calculated using Gray's test.

b *P*‐Value for each covariate for risk of CBC, calculated using Gray's test.

### 3.4 Influence of nuclear grade and number of blocks with positive sites on IBTR‐TR

Of 46 margin‐positive DCIS, 9 cases developed IBTR‐TR during the median follow‐up period of 7.6 years. We explored the influence of nuclear grade and the number of blocks with positive margins on IBTR‐TR. First, the higher the nuclear grade, the more frequent the IBTR‐TR (Table 5). Only 9% of low/intermediate nuclear grade DCIS developed IBTR‐TR, whereas 50% of high nuclear grade DCIS developed IBTR‐TR. The majority (7/9) of IBTR‐TR occurred in intermediate‐ or high‐grade DCIS that had more than 2 blocks with positive sites. In summary, the risk of IBTR‐TR was low if the nuclear grade was low or intermediate and the positive sites were localized in a single block. In contrast, the risk of IBTR‐TR was high if the nuclear grade was high, even if the positive sites were localized in a single block.

**Table 5:** Number of paraffin blocks with positive sites, DCIS grade, and IBTR‐TR

Grade	No. of blocks with positive sites	No. of cases	IBTR‐TR
Low	1	11	1
	2	6	0
	3	4	0
Intermediate	1	5	0
	2	5	1
	3	3	1
High	1	3	1
	2	6	3
	3	3	2

Note

DCIS, ductal carcinoma in situ; IBTR‐TR, ipsilateral breast tumor recurrence‐true recurrence (IBTR with a positive margin by the polygon method at the primary BCS).

## 4 DISCUSSION

In DCIS of the breast, neoplastic epithelial proliferation is usually confined to a single segment of the ductal‐lobular system.^25^, ^26^ With the advent of high–quality mammography in routine clinical practice and breast cancer screening programs, the incidence of small and unifocal DCIS is steadily increasing. Currently, such DCIS is often treated with BCS. However, performing adjuvant RT uniformly in DCIS patients would result in overtreatment of those who have no remaining DCIS in the preserved breast. This study was performed to clarify whether the polygon method, a systematic method of en face margin assessment, can identify a subset of DCIS that can be safely treated by BCS alone, at least in Japanese women.

Our approach differs from conventional margin evaluation by several points. First, in the conventional approach, breast excision margins are evaluated on sections cut perpendicular to the outer surface of the specimen after painting the surface with insoluble dye.^18^, ^27^, ^28^ A major limitation of this approach is its ineffectiveness in determining whether the entire DCIS is completely removed because DCIS spreads insidiously along the three‐dimensional mammary duct lobular system, resembling the root of plant^29^; whereas the inked margin only reveals a two‐dimensional cross‐section of the system. Therefore, a mere negative finding by the conventional method (inked margin) does not exclude the remaining DCIS in the preserved breast. According to the Institute Curie Breast Study Group who studied the predictive value of conventional inked margins in lumpectomy for DCIS, there was residual tumor in 44% and 45% of close noninvolved (>1 and ≦1 mm width, respectively) margins.^16^ In contrast, the advantage of the en face approach is that it permits evaluation of a much greater extent of the surface of the specimen with fewer blocks than the conventional perpendicular method of margin assessment. This current study demonstrated that in 80% of cases, the number of blocks required for en face margin evaluation was 8 or fewer. Second, we resolved a preanalytical issue that may influence the accurate orientation of the critical margin. This phenomenon is difficult for pathologists or pathology assistants to identify because this deformity has already occurred before the specimen arrives at the pathology laboratory. Graham et al stated that breast specimens lose almost 50% of their original height after surgical removal.^30^ To address this issue, we developed a systematic method with an adjustable mold to prevent the “pancake phenomenon”. Third, we focused on the entire vertical cut edges of the specimen. The margin that pathologists are primarily responsible for in wide local excision is the interphase between the preserved and the removed tissue rather than the deep (pectoral muscle fascia) and the superficial (skin) margins. Specific to the wide local excision of the breast is the peripheral margin: the deep and the superficial margins of our wide local excision are the almost same as that for mastectomy. Thus, this new method makes it possible to observe a panoramic view of the entire peripheral margin of the wide local excision.

The main objective of this retrospective cohort study was to reveal the clinical outcomes of Japanese women with DCIS treated with BCS alone when the negative margin was confirmed by systematic en face margin evaluation using the polygon method. Through this approach, 100 patients (69%) were identified to have negative margins. Of these 100 women, 5 developed IBTR and 10 developed CBC during a median follow‐up period of 7.6 years. Competing risk analysis in the subset of women with negative margins as determined by the polygon method indicated that 5‐ and 10‐year rates were 3.0% and 5.3% for IBTR, and 7.1% and 13.3% for CBC, respectively. Thus, IBTR was consistently at least twofold less than CBC risk despite no patients in this subset of patients receiving adjuvant radiotherapy. These results support our hypothesis that IBTR only contains NP if the margin evaluated by the polygon method is negative. When IBTR risk is less than or equal to the risk of developing CBC, omission of RT in this subset would be a reasonable approach.^31^ RT is expensive and time‐consuming, and may be accompanied by serious side effects. Furthermore, it is beneficial for patients with DCIS when keeping the treatment option of RT in reserve for potential more serious situations in the future, such as recurrence at the chest wall or the axilla. Avoiding unnecessary RT is important because tissue can only receive a limited amount of radiation before it is permanently damaged by radiation. Our data indicate that DCIS patients with a negative margin identified by the polygon method are candidates for BCS alone without RT.

On univariate competing risk analysis, the risk of IBTR was significantly higher when the index DCIS was diagnosed at a younger age, presented clinically, and of large size or of 3+ HER2 status, consistent with previous studies.^32^, ^33^ However, the lower rates of IBTR compared with CBC after BCS for DCIS patients whose margin was negative by the polygon method observed in our study are in sharp contrast with those in prior literature that consistently showed higher rates of IBTR than CBC following BCS with or without radiotherapy. According to the National Surgical Adjuvant Breast and Bowel Project study reported by Fisher et al, in the lumpectomy alone group, 16.3% (64/391) women developed IBTR whereas 2.0% (8/391) women developed CBC. In the group treated by lumpectomy and radiation, 7.0% (28/399) women developed IBTR whereas 2.5% (10/399) women developed CBC.^10^ In 129 DCIS studied by Holland et al, IBTR was 9.3% (12/129) whereas CBC was 0.8% (1/129).^34^ Recently Miller et al studied women with DCIS treated with BCS at Memorial Sloan Kettering Cancer Center from 1978 to 2011. Of 2,759 patients with DCIS treated with BCS, 344 developed IBTR and 151 developed CBC. On competing risk regression, 10‐year IBTR and CBC rates were 14.5% and 5.8%, respectively. Overall, 10‐year IBTR rates were 2.5‐fold higher than CBC rates, and, without radiation, fourfold higher.^35^ Although the mechanism for higher rate of IBTR compared with CBC after BCS for DCIS in previous studies has not been fully discussed, we speculate that IBTR in studies using conventional margin evaluation include many TR, namely regrowth from the DCIS missed by inked margin assessment. For a relatively high contralateral breast cancer risk in this study, we considered the following 3 points. First, an established risk factor for future development of breast cancer was removed from the ipsilateral breast by BCS. Second, background nonneoplastic breast tissue, including remaining ipsilateral and contralateral breast, may be at general risk of future breast cancer development. Third, at our institution, ultrasonography and, if necessary, MRI, in addition to mammography, are used in the annual follow‐up of patients with BCS. This may increase the sensitivity of breast cancer detection. Furthermore, the sensitivity of ultrasonography and mammography for lesions in the contralateral breast may be higher than those in the ipsilateral breast because the former can be compared with its past image with no surgical scar.

Using the sequential slicing method, Silverstein et al reported that DCIS cases with negative margins as assessed by meticulous sampling techniques showed very much lower local recurrence rates after BCS alone for DCIS of the breast.^36^ This study is a pioneering work ensuring the local cure of DCIS by surgery alone. The main difference between Silverstein's approach and ours is that our system is a combination of the peripheral en face sectioning method and the sequential slicing method. In our system, the critical peripheral margin is assessed by the sensitive en face margin. In addition, the width of our slice is 5 mm, whereas theirs is 2.5 mm. In Silverstein's system, the margin evaluation is perpendicular to the surface, as is conventional margin evaluation. The accuracy of perpendicular margin evaluation depends on slice pitch and margin width, but en face margin evaluation is independent of these. To increase the accuracy of the inked margin method, laborious and time‐consuming analysis of histological sections is necessary. One advantage of the polygon method is that the accuracy of margin evaluation is not affected by the sampling for tumor characterization. This may be important when our system is applied to BCS for IDC.

It is interesting to understand how DCIS cases with positive margins as determined by the polygon method would be judged using the conventional inked margin method. Guidi et al studied 22 consecutive breast reexcision specimens in which the specimen surfaces were inked, margins were shaved, and tumor was present in at least one of the shaved margin sections.^23^ Among 69 positive shaved margins, the corresponding inked margin was positive in only 42 (61%). The results of Guidi's study suggest that 40% of DCIS cases with a positive margin according to the polygon method would be negative by the inked margin approach. This calculation may explain the high IBTR rates over CBC after BCS despite “negative margins” in the randomized control trials.

This study has several limitations. First, all patients were Japanese, whose breasts characteristically tend not to be voluminous. It remains to be determined whether our approach is applicable to women of other ethnic groups. When our method is applied to other ethnic groups whose breasts are larger than that of the Japanese population, the size of the mold should be modified. Second, critical margins differ from the periphery when the breast lesion is located just beneath the nipple. In this case, the main ducts rise to the nipple. Therefore, the shaved margin should be obtained from the superficial margin. Third, although our method is applicable to BCS for phyllodes tumor and IDC, further clinicopathological validation is necessary especially before applying this new approach to the BCS for IDC. In particular, data addressing the influence of lympho‐vascular invasion on IBTR, should be collated.

In conclusion, our retrospective cohort study indicates that women whose excision specimens are classified as having a negative margin by the polygon method show a very low risk for IBTR, accounting for approximately half of CBC cases. In this subset of DCIS, adjuvant RT is not beneficial. To the best of our knowledge, this paper is the first to report the impact of systematic en face margin assessment on IBTR of DCIS patients undergoing BCS. Our results lead to the hypothesis that the rate of IBTR and CBC reflects the number of TDLUs in the affected and healthy sides after the index DCIS is completely removed. Although it may look simple, our approach requires a close collaboration between the breast team, including radiologists, surgeons, and pathologists. The team should reach a consensus with regard to the concept of the new margin evaluation and should fully understand their roles in undertaking the new strategy. In our breast team, clinicians who are specialized in breast imaging draw the tumor distribution map on the patients’ skin prior to surgery. After surgery, the surgeon places the specimen in the adjustable mold because they are the best person to know the orientation and direction of the specimen.

## CONFLICTS OF INTEREST

The corresponding author has patents of tools relevant to the work. Otherwise, authors have no conflict of interest to declare.

## AUTHOR CONTRIBUTIONS

Shu Ichihara: Conceptualization, formal analysis, funding acquisition, software, writing—original draft, and writing—review and editing. Suzuko Moritani, Rieko Nishimura, Mikinao Oiwa, Takako Morita, Takako Hayashi, Aya Kato, Tokiko Endo, Akiko Kada, Noriko Ito, Tetsuo Kuroishi, Yasuyuki Sato: Data curation, methodology, and writing—review and editing.

## Data Availability

The data that support the findings of this study are available from the corresponding author, SI, upon reasonable request.
